# Universal diamond edge Raman scale to 0.5 terapascal and implications for the metallization of hydrogen

**DOI:** 10.1038/s41467-023-36429-9

**Published:** 2023-02-17

**Authors:** M. I. Eremets, V. S. Minkov, P. P. Kong, A. P. Drozdov, S. Chariton, V. B. Prakapenka

**Affiliations:** 1 Max Planck Institute for Chemistry, Hahn Meitner Weg 1, Mainz, 55128 Germany; 2 Center for Advanced Radiation Sources, University of Chicago, 5640 South Ellis Avenue, Chicago, IL 60637 USA

**Keywords:** Structure of solids and liquids, Chemical physics, Structure of solids and liquids

## Abstract

The recent progress in generating static pressures up to terapascal values opens opportunities for studying novel materials with unusual properties, such as metallization of hydrogen and high-temperature superconductivity. However, an evaluation of pressure above ~0.3 terapascal is a challenge. We report a universal high-pressure scale up to ~0.5 terapascal, which is based on the shift of the Raman edge of stressed diamond anvils correlated with the equation of state of Au and does not require an additional pressure sensor. According to the new scale, the pressure values are substantially lower by 20% at ~0.5 terapascal compared to the extrapolation of the existing scales. We compare the available data of H_2_ at the highest static pressures. We show that the onset of the proposed metallization of molecular hydrogen reported by different groups is consistent when corrected with the new scale and can be compared with various theoretical predictions.

## Abstract (web summary)

The progress in generating high static pressures in diamond anvil cells opens opportunities for studying novel materials with unusual properties. Here, the authors report a universal high-pressure diamond edge Raman scale up to 500 gigapascals, which does not require an additional pressure sensor.

## Introduction

The terapascal pressure range has recently been achieved in dynamic compression experiments at large facilities^1^. Amazingly, similar pressures can be generated in static experiments using the incommensurably smaller and simpler device – a diamond anvil cell (DAC)^2,3^, appropriate for many more diversified studies. Under such extreme pressure conditions, the structure and properties can change drastically even at ambient temperature because the work associated with the *P*–*V* compression is comparable to the energy of atomic bonds. For example, the “simple metal” sodium transforms to an insulator (electride)^4^; conversely, oxygen and xenon transform to metals^5,6^. Molecular nitrogen transforms into a single-bonded state with a diamond-like structure^7^.

Motivation for generating multi-megabar static pressures is primarily driven by the pursuit of obtaining atomic metallic phase of hydrogen, which is predicted to be a room temperature superconductor at pressures of ∼500 GPa and may combine superconductivity and superfluidity^8^. There has been substantial progress in the study of hydrogen, both theoretically and experimentally. Electrical conductivity studies indicate that molecular hydrogen metalizes at ∼320 GPa through the closing of the indirect gap^9–11^. At higher pressures, a direct bandgap likely closes according to the abrupt decrease in the infrared absorption at ∼427 GPa^12^ and disappearance of the Raman modes at ∼450 GPa^10^. The problem of metallic hydrogen, in turn, has stimulated the search for high-temperature superconductivity in hydrogen-rich compounds. Nearly room temperature superconductivity with a *T*_*c*_ of ∼203 K in H_3_S^13^ and *T*_*c*_ ∼250 K in LaH_10_^14,15^ was reached at pressures of ∼150–170 GPa. The latest theoretical calculations predict *T*_*c*_s exceeding room temperature: *T*_*c*_ ∼330 K in CeH_18_^16^, and ∼470 K for Li_2_MgH_16_^17^ at higher pressures of ∼300–500 GPa.

Static pressures up to 1 TPa were gradually increased starting from the modest ∼10 GPa by improving the diamond anvil geometry and DAC design. A decisive step was the development of beveled anvils^18^, which instantly opened the way for pressures beyond 100 GPa. With more complicated profiling of anvils, such as toroidal grooves, which surpass the plain Bridgeman anvils^19,20^ pressures of ∼400 GPa^2,12^ up to a maximum of ∼600 GPa^2^ were achieved. Encouragingly, ∼1 TPa was reached between two tiny hemispherical nanocrystalline diamonds interposed between conventional diamond anvils^3^.

However, determining the pressure inside a DAC is difficult. The absolute values of pressure can be derived from a combination of *V(P)* data measured by X-ray diffraction and derivative d*V*/d*P* data measured by ultrasonic or Brillouin scattering, but it is limited to 120 GPa^21^. The pressure estimation in DACs has been based on temperature-corrected shockwave compression data^22^. In contrast to adiabatic compression in shock waves, the recently developed shockless ramp technique provides nearly isentropic conditions and lower heating effects. According to ref. ^23^ the pressure along the isentrope at 600 GPa differs by only ∼6 GPa compared to the 298 K isotherm. The isotherms of copper, gold, platinum and other materials^23^ can be used for pressure estimation in DACs; however, a synchrotron X-ray source is needed to probe the lattice volume of these standards.

Instead, more convenient secondary scales are widely used, such as the ruby luminescence scale calibrated to 156 GPa against the equation of state of some metals^24^. However, the applicability of the ruby scale is limited to ∼200 GPa because of the drastic weakening of the ruby luminescence^25^.

**Fig. 1:**
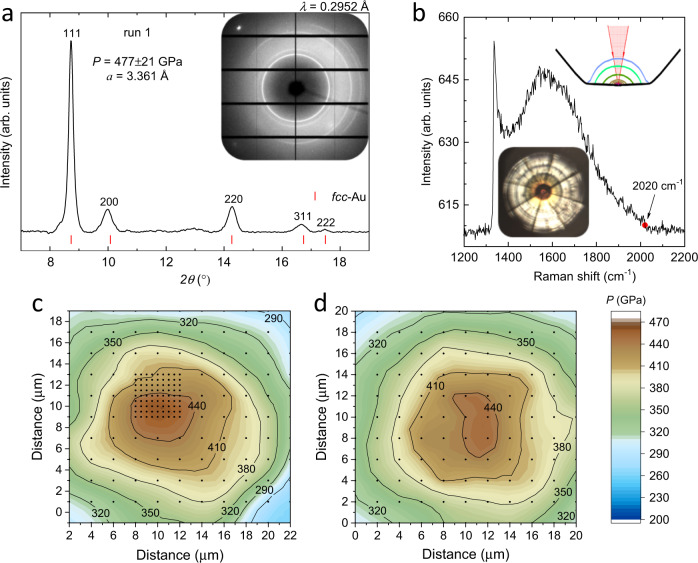
X-ray diffraction and Raman measurements for the diamond edge Raman scale. **a** X-ray powder diffraction pattern of gold in run 1 at the maximum pressure value of 477±21 GPa. Red ticks indicate the calculated positions of gold. The inset shows the original diffraction pattern. **b** Raman spectrum recorded in run 1 at the same pressure. The Raman band (1333 cm^−1^–2020 cm^−1^) is formed by the response of the whole stressed volume of the diamond anvil starting from the signal of the unstressed diamond inside the anvil to the signal of the diamond culet, where the Raman shift is maximum. It has a cutoff in form of step, a midpoint at the step is at ∼2020 cm^−1^. The signal is excited by the focused laser (red arrows) as shown at the top inset. This insert shows the profile of the diamond anvil (black line), and the contours show the distribution of the pressure inside the anvils as it calculated for 280 GPa in refs. ^28,29^. The fringes in the spectrum are discussed in Supplementary Information. The lower inset shows the photo of the sample at 477±21 GPa. **c**, **d** Spatial distribution of pressure on the diamond tip in run 2 reconstructed from the X-ray powder diffraction data using a gold equation of state^23^ (**c**) and from Raman spectroscopy data of the stressed diamond anvil (**d**). Black points are spots of measurements. The pressure values estimated by two different techniques agree well. Source data are provided as a Source Data file.

The only practical alternative to X-ray probes for pressure determination above ∼200 GPa is the diamond edge Raman scale. It is based on the fact that a Raman spectrum measured from a sample in a DAC inevitably contains a signal from the stressed anvils – a strong, broad band with a well-defined cutoff (Fig. 1b and Supplementary Fig. 1 and refs. ^25,26^). This high-wavenumber edge correlates with the pressure in the sample. The band at lower wavenumbers stems from the Raman signal of the diamond at deeper regions, where stresses are lower. We note that pronounced fringes are often observed within this band, their origin is discussed in Methods. Hanfland and Syassen^26^ analyzed the Raman spectra of stressed anvils and proposed a linear pressure dependence of the diamond Raman edge up to 30 GPa. A linear pressure dependence with different constants was also suggested up to ∼200 GPa^27^. However, the linear pressure dependence cannot be used as a practical scale with a sufficiently high precision. As noted in ref. ^26^, the stress pattern within the strained diamond anvil differs from the considered uniaxial case^26^, which is highly anisotropic and possibly depends on the geometry of the diamond anvils and gasket material, pressure medium, and/or sample stiffness. Recent comprehensive calculations by V. Levitas et al.^28,29^ provided likely a realistic complex picture of stresses and strains which develop in anvils at 400 GPa. These results can, in principal, be used as a basis for calculation of a Raman spectrum of the stressed anvils. Perhaps the reverse task – the simulation of stresses from the measured Raman spectrum can be solved too. Such study would be valuable for understanding the limitation in a pressure for already used shapes of anvils and would provide a clue for further developments towards the higher pressures. The optimal shape of a diamond anvil suitable for a pressure above ∼500 GPa still has not been established. The toroidal anvils likely have advantages because the highest pressure of ∼600 GPa was documented^2^, however, it succeeded only once. The subsequent reported results on toroidal anvils showed pressure values slightly above 400 GPa^12^, which are comparable with those achieved in traditional double-beveled anvils as demonstrated in the present paper.

The actual diamond pressure scale remains to be empirical – it was established based on numerous experiments at pressures up to 200 GPa with a ruby chip and X-ray pressure sensors^9,25^. It was found that the nonlinear pressure-induced shift of the diamond Raman edge is surprisingly suitable for a reliable estimation of pressure in diamond anvils of different shapes, with various gaskets and samples^25^. Akahama and Kawamura^30,31^ extended the diamond pressure scale to 410 GPa by correlating the diamond Raman edge with the X-ray diffraction data of a Pt sample. However, the pressure estimation above ∼250 GPa has since remained uncertain, particularly because the extended scale^31^ apparently deviates from the extrapolation of the well-established low-pressure data^25,30^ reaching a ∼10% difference at ∼400 GPa. The authors^31^ suggested that the reason for the puzzling deviation might be a certain change in the stress state on the culet face of diamond anvils or the accuracy of the equation of state of Pt used^32^. However, the updated equation of state of Pt did not overcome this contradiction^23^. One can speculate that the Raman signal might be picked up not at the maximum pressure, e.g. out of the anvil surface, in a deeper inside. In this case the cut-off at the Raman spectrum would give systematically lower values. An overestimation of pressure values in Akahama scale^31^ has been suspected by different groups^12,33,34^ based on abnormal deviation of the loading curve^12^ and the pressure dependence of the frequency of hydrogen vibron above ∼300 GPa^12,33^. The estimation of pressure above 400 GPa is even more uncertain because extrapolations of different scales yield unacceptably large deviations up to ∼100 GPa at 500 GPa^9,25,30,31^.

The present study establishes a reliable diamond-edge Raman scale at pressures up to ~500 GPa. The accurate determination of pressure values in the 250–500 GPa pressure range is crucial because of the recent significant progress in the generation of extremely high static pressures, the study of metallic hydrogen and high-temperature superconductivity, phase transitions, chemistry, and the necessity of comparison between experiments of different groups and theoretical predictions, which are becoming more and more precise.

## Results

### Raman shift of stressed diamond anvils vs equation of state of gold

**Fig. 2:**
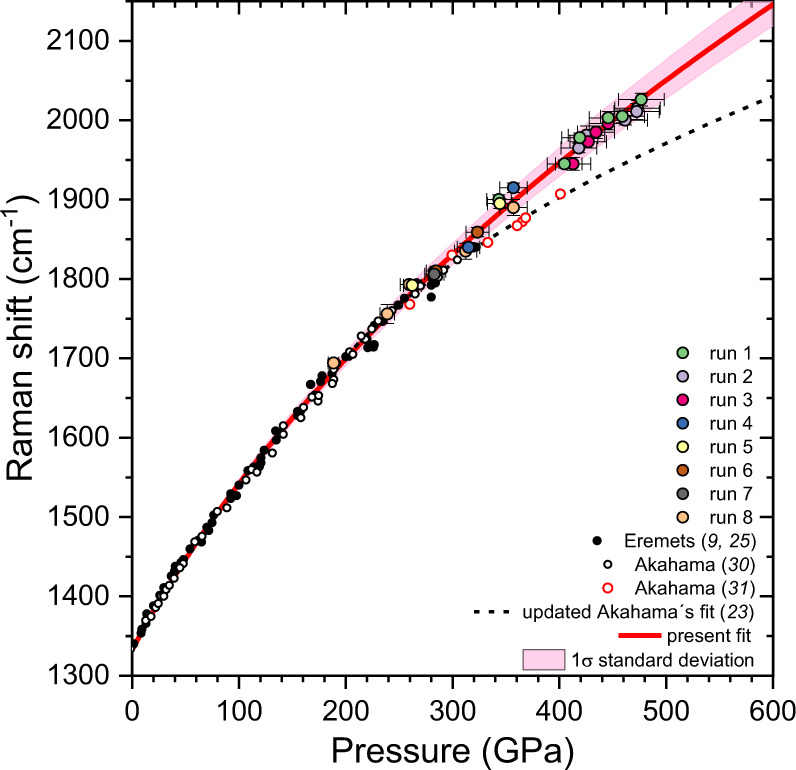
The universal diamond edge Raman scale. Large colorful circles correspond to the pressure dependence of the high-wavenumber Raman edge of the stressed diamond anvil measured in runs 1–8 of the present study. Error bars represent uncertainties in estimation of pressure values using equation of state of Au^23^ and determination of the mid-point of the diamond high-wavenumber Raman edge. Small black circles are the data from refs. ^9,25^ (pressures values were estimated using the ruby scale^24,25^ below ∼200 GPa and equation of state of Au^63^ above ∼200 GPa). Open black and red circles correspond to the data from refs. ^30,31^ after correcting the pressure values using the recent ramp compression data of Pt^23^. The solid red curve fits the combined experimental data set, including the previous data measured below ∼300 GPa^9,25,30^, and magenta area shows 1σ standard deviation (see Supplementary Table 1). The black dotted curve is the updated diamond edge Raman scale of Akahama^23^ for the high-pressure range above 200 GPa. Source data are provided as a Source Data file.

We prepared eight DACs with a gold sample as the X-ray pressure standard. The single-crystal diamond anvils had a culet plane normal to the [001] crystallographic direction. We created profiles of anvil tips of different shapes to achieve the highest pressure (see Supplementary Fig. 2). We established the correlation between the pressure values, which were estimated from refined lattice parameters of a gold sample probed with X-ray diffraction, and the high-wavenumber Raman edge of stressed diamond anvils from the same point of the sample (see Fig. 1). The equation of state of gold was taken from the isotherm derived from the ramp data^23^ (see Methods for a detailed description of the experiment). We plotted this correlation over a wide pressure range for anvils of different shapes. The data are consistent with each other in different runs and with previously reported data obtained below ∼300 GPa^9,25,30^ (see Fig. 2). We fitted the combined data set over the entire pressure range of 0–477 GPa using equation proposed in ref. ^30^, $P=A\cdot \frac{\triangle \omega }{{\omega }_{0}}+B\cdot {(\frac{\triangle \omega }{{\omega }_{0}})}^{2}$, where Δω is the shift of the diamond Raman edge of the stressed anvil, *ω*_0_ = 1332.5 cm^−1^ is the initial position of the diamond Raman edge of unstressed diamond anvils at ambient pressure, and *A* and *B* are the fitting parameters. The refined parameters *A* = 517 ± 5 GPa and *B* = 764 ± 14 GPa fit the data well and apply for accurate pressure estimation from ambient pressure to ∼500 GPa.

The new diamond scale contradicts the currently used diamond scale^31^ at pressures above 250 GPa, reaching a difference in pressure values over 100 GPa at approximately 500 GPa (see Fig. 2). The highest pressure of 477±21 GPa based on a gold equation of state^23^ measured in the present study corresponds to the record highest shift of the diamond Raman edge of stressed anvils of ∼2026 cm^−1^ (∼592 GPa according to the old scale^31^). It was achieved in run 1 using toroidal diamond anvils with culets of diameter ∼10 µm. Note that a similar pressure of 446±19 GPa (the diamond Raman edge at ∼1996 cm^−1^) was reached in run 3 with traditional, not toroidal, but double-beveled diamond anvils (a culet size of 12 µm).

The diamond edge Raman scale was robust to different load distribution patterns on the diamond tip. It works well for diamond anvils of different shapes (see Supplementary Fig. 2). Notably, the refined diamond scale is valid for pressure estimation at the center of the diamond anvils and for a large area of ∼20–30 µm around the diamond tip (see Fig. 1d and Supplementary Fig. 3 – 5).

**Fig. 3:**
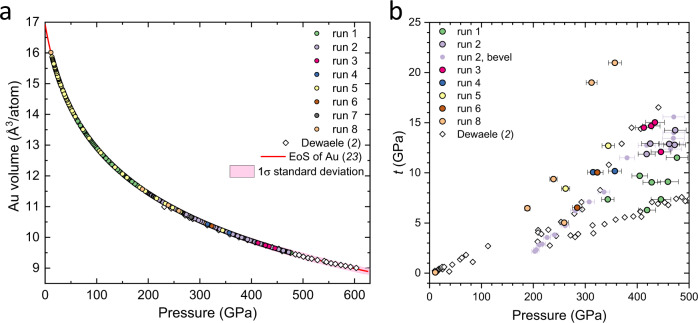
X-ray diffraction measurements for the diamond edge Raman scale. **a** Lattice volume of gold vs pressure measured in runs 1–8. Pressure values were estimated from the isotherm obtained from the ramp compression^23^. Magenta area corresponds to 1σ standard deviation. **b** Uniaxial stress *t* sustained by gold samples in runs 1–6 and 8. Large colorful circles with black edges correspond to values of uniaxial stress calculated at the highest pressure in each compression cycle. Small plain circles correspond to values of uniaxial stress in the gold sample in run 2 at different spots by moving from the center of diamond culet to the bevel (the total length of ∼39.6 μm, the step of ∼2.8 μm). Error bars show uncertainties for estimated pressure values from X-ray diffraction data using equation of state of Au^23^. Open black rhombuses are the data from ref. ^2^ for comparison. Source data are provided as a Source Data file.

It is worth noting that high pressures conditions produce the non-hydrostatic stress on the compressed sample. A soft “quasi-hydrostatic” pressure-transmitting medium is usually used to minimize the effect of uniaxial stress. However, gold samples sustain considerable non-hydrostatic stress already at ∼100 GPa even being embedded in helium medium^35,36^, resulting in the anomalous shift of the (200) diffraction peak. In the absence of soft medium, the yield stress of the gold itself limits the non-hydrostatic stress and subsequent stress gradients. We estimated the non-hydrostatic deviatoric stress *t* in Au samples in our experiments to get a further insight to reliability of the collected data (see Fig. 3). The estimated values of *t* reaches ∼10–15 GPa at the highest pressures in most our runs. These values are comparable with those estimated for Au samples compressed up to ∼600 GPa in toroidal diamond anvils^2^ and lower than values estimated in the ramp experiments^23^.

### The universal diamond edge Raman scale and hydrogen vibron

The universal scale for a sample pressure estimation is crucial for collating experimental results from different groups and comparison with theoretical predictions. Because of the inconsistency in the pressure scales used in the reported data, the intriguing observations of pressure-induced phase transitions in hydrogen and its metallization are difficult to compare in the multi-megabar pressure range. Before the present study, there was no consensus on which pressure scale should be used. The latest diamond edge Raman scale calibration up to 410 GPa^31^ and more conservative scale^30^ both were used^12,33,34^ to study hydrogen, but they differ dramatically above ∼300 GPa. This ambiguity raises the question of how suitable the diamond edge Raman scale is for hydrogen. The diamond scales are based on the equation of state of metals, but hydrogen is much softer. In addition, the compression of hydrogen is sensitive to the thickness and diameter of the sample and material of the gasket because a particular arrangement of experiments influences the stresses in the diamond anvils and the Raman spectrum. In particular, it develops in a pronounced sharp peak at the high-wavenumber Raman edge of diamond anvils recorded over the hydrogen sample (see Supplementary Fig. 1, run 7), whereas it manifests as a weak step in Raman spectra recorded over gold samples (other runs in Supplementary Fig. 1).

We checked the validity of the diamond edge Raman scale by measuring the lattice volume of gold placed inside the hydrogen sample in run 7. Gold is the only metal that likely does not react with hydrogen. Theoretical works are contradictory: Kim et al.^37^ predicted the formation of AuH above 220 GPa, whereas Gao et al.^38^ demonstrated that AuH is thermodynamically unfavorable against elements. Experimentally, no hydrides of gold have been found at pressures up to 113 GPa and temperatures up to 600 K^39^. In the present experiments performed at room temperature, gold did not form a hydride up to the highest pressure of ∼290 GPa, as both the face-centered cubic crystal structure and the lattice volume of the sample corresponded to pure gold^2,3^. The pressure estimated from the gold sample surrounded by hydrogen in run 7 is in good agreement with the pressure value simultaneously estimated from the new diamond Raman edge scale, which strongly supports the application of the updated diamond edge Raman scale to hydrogen samples.

**Fig. 4:**
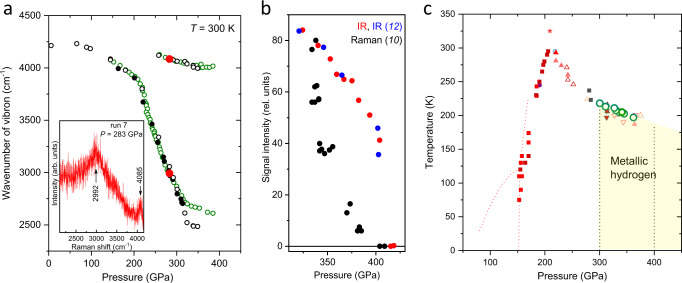
Comparison of different experimental studies of hydrogen at high pressures. **a** Pressure dependence of the hydrogen vibrons at room temperature. Solid black, open black, and open gray circles correspond to data from ref. ^9^ Open green points are from ref. ^33^; in this work, the pressure scale^30^ was used, which is close to the present scale in this pressure range. The pressure for the rest was determined from the present diamond edge scale. Red circles are the data measured in run 7 of the present study. The open circles were shifted to lower pressures by 17 GPa to be consistent with the wavenumber of hydrogen vibron at the calibrated point (red circles). **b** Comparison of the infrared absorption data^12^ (blue circles) and the Raman data^10^. Pressure values for infrared and Raman data were recalculated according to the present scale (the values were shifted by ∼10 GPa to lower pressures for infrared measurements^12^; the maximum pressure of 475 GPa was significantly lowered to 409 GPa for Raman data^10^. The comparable updated pressure values for the infrared and Raman data likely indicate the same transition. **c** The updated phases diagram of hydrogen according to the present pressure scale. The color points and curves are from ref. ^10^, green circles are from ref. ^11^ Vertical dotted black line at 300 GPa indicates the boundary of the metallic state of hydrogen (closure of the indirect gap), and the line at 400 GPa indicates a possible structural or electronic transition to phase VI^10,12^. Source data are provided as a Source Data file.

The position of the hydrogen vibron in the Raman spectra is sensitive to pressure. For example, one can observe a large scatter in the data of ~20 GPa from run to run on the plots of the pressure dependence of hydrogen vibron^9,40^. The accuracy of the pressure determination can be significantly improved by using hydrogen itself as a pressure gauge by calibrating the pressure-induced shift of hydrogen vibron *ω(P)* against the gold chip placed in hydrogen. We found that hydrogen vibrons at ∼2992 cm^−1^ and ∼4085 cm^−1^ correspond to a pressure of 283 ± 9 GPa, as determined from the gold chip (see the inset in Fig. 4a). At higher pressures, diamond anvils broke during exposure to intense synchrotron X-rays; this premature failure is common^41^. This calibration of the hydrogen vibron can serve as a reference, and the pressure correction can be performed by shifting the whole plot of *ω(P)* to the calibrated point at *P* = 283 ± 9 GPa, as shown in Fig. 4a.

### High-pressure phase diagram of hydrogen

The phase diagram of hydrogen was corrected according to the updated universal diamond scale (see Fig. 4c). The phase diagram is built on experimental data from different groups^9,33,42,43^ with the primarily goal to locate the area of metallic hydrogen. According to the current conception hydrogen transforms from dielectric and semiconducting state into a semimetal at ∼300 GPa^11^ (see Fig. 4c). This follows from the well reproducible measurements on the temperature dependence of electrical conductivity^9–11^. These experiments are well supported by current theoretical calculations showing that at pressure of ∼350–400 GPa the indirect gap of hydrogen closes, free electrons and holes appear indicating that hydrogen turns into a metal^44–47^. After the overlap, the density of states around the Fermi level is low indicating the formation of a bad metal with properties similar to a semimetal^44,45^. The closure of the indirect gap, however, is difficult to detect by infrared (IR) absorption measurements in DACs because the magnitude of the change in absorption is too small (the optical transitions are between different points of the k-space – indirect transitions)^44,45^ and a thickness of the sample is only few micrometers. On the other hand, the weak absorption allows to observe the Raman signal in the semimetallic state of hydrogen^10^. Upon increase of pressure, the overlap of electronic bands increases, hydrogen turns into a better metal^44,45^, the absorption increases and the Raman signal vanishes^10^ (see Fig. 4b).

The IR absorption measurements which are insensitive to detect the transition into a semimetal at ∼300 GPa revealed the sharp decrease in IR absorption at ∼427 GPa, that was interpreted as a structural transition to a good metal^12^. Further evidence of the metallic state of hydrogen from electrical transport measurements is required. Most likely hydrogen remains in the molecular state as the reverse transition occurs at the same pressure (a first order phase transition accompanied by dissociation/association of hydrogen molecules must exhibit a pronounced hysteresis in pressure). The disappearance of the Raman signal was reported at similar pressure values of ∼450 GPa^10^. Taking into account the new corrected pressure scale, both observed transitions are, in fact, at close pressures of ∼400–410 GPa (see Fig. 4b), indicating that both observations are probably related to the same transition. Experimentally, it is difficult to distinguish whether this transition is structural or electronic. Theoretically, the structural *C*2/*c*-24 to *Cmca*-12 phase transition is predicted in molecular hydrogen at ∼420 GPa^47,48^. The alternative scenario implies the closure of the direct band in hydrogen and its transformation into a good metal at ∼450 GPa^44,45^ or ∼430 GPa^46^ within the same *C*2/*c*-24 molecular phase. This metal is predicted to be a superconductor with a *T*_*c*_ ∼86 K at 400 GPa arising to ∼212 K at 500 GPa^49^. More experimental data are required to understand the nature of the reported observations.

At higher pressures, hydrogen should dissociate into atomic state – an ultimate goal of the studies of metallic hydrogen initiated by Wigner and Huntington^50^. Recent calculations place this transition into Cs-IV phase at 447 GPa^48^ or at ∼577 GPa^47^. Experientially, the metallization of hydrogen was claimed by Dias and Silvera^34^ at 495 GPa, it was based on visual observations and reflection measurements by two wavelengths. In view of the new scale, the pressure of metallization slightly shifts to 504 GPa. Such pressure seems to be unrealistically high, i.e. much higher than the highest pressure values of ∼400–420 GPa^10,12,51^ ever reached for hydrogen samples clamped in either conventional bevelled or toroidal diamond anvils of even much smaller size of culets^52^. This contradictory claim^34^ was met with serious criticism^53^, and since then it has neither been reproduced nor confirmed.

Summarizing up, we note that the correct pressure evaluation is crucial for the interplay between theory and experiment, which can be very fruitful, as illustrated by recent progress in near-room temperature superconductivity. The experiment, which is often guided by calculations, provides discrepancies that improve the computational models and fundamental understanding. Reliable predictions of crystal structures and properties of matter at high pressures, together with the development of the synthesis of new compounds, will advance science and technology.

## Methods

### Diamond anvil cell preparation

High pressure was generated using DACs with a diameter of 25 mm and a length of 35 mm. The load is applied by pushing the piston, which is moved by screws at the top of the DAC. Anvils are made of synthetic or natural diamonds with culets that are normal to the [001] crystallographic direction. They were beveled at ∼8° to a diameter of ~250–400 µm. We used small culets of ∼8–20 µm with different shapes of the diamond tip to achieve the highest pressures (see Supplementary Fig. 2). The toroidal shape of the diamond anvils was made with the aid of a focused beam of xenon ions (FERA3, Tescan). The ion beam current was set between 1 and 10 nA under an accelerating voltage of 30 kV. The total duration of machining of the toroidal shape was approximately 1 h per diamond anvil. The final profiles of the diamond anvils was measured using a Profilm3D optical profilometer from Filmetrics (white light interferometry; a roughness of 0.05 μm). For the sample loading, 250-µm-thick T301 stainless steel gaskets were pre-indented to a thickness of ∼5 µm, and a hole with a diameter of ∼20 µm was drilled by a laser. A piece of gold (SkySpring Nanomaterials, 99.99%) was placed in the prepared hole and clamped in the DACs. Gold was used as the standard for the pressure determination. In total, eight DACs were pressurized to ∼200–300 GPa in the home laboratory and transferred to synchrotron facilities, where the pressure was further increased. Several photos of diamond anvils with gold samples at highest achieved pressures in runs 1–3 are shown in Supplementary Fig. 6.

### X-ray diffraction and Raman measurements

X-ray diffraction data were collected at the beamlines 13-IDD at GSECARS, Advanced Photon Source (*λ* = 0.2952 Å, a spot size of ~2.5 × 3.5 µm^2^, Pilatus 1 M CdTe detector) and P02.2 at PETRA III, DESY (*λ* = 0.2910 Å, a spot size of ~3 × 3 µm^2^, LAMBDA GaAs detector). The typical exposure time was varied between 1 s and 5 s. Reference samples of LaB_6_ and CeO_2_ were used to calibrate the distance between the sample and detector. The X-ray beam was cleaned using a pinhole to remove the beam wings. The processing and integration of the data and background subtraction were performed using the Dioptas software^54^.

The Raman spectra were recorded using an advanced Raman setup at GESECARS^55^. The Raman signals of stressed diamond anvils in contact with gold samples were excited by lasers with two different wavelengths: *λ*_1_ = 660 nm and *λ*_2_ = 532 nm. The switching from red to green laser helped to reduce and shift the strong luminescence of the stressed diamond, which may appear in synthetic diamonds at pressures above 300 GPa and overlap the diamond Raman edge signal of stressed anvils. The laser power of the incident light coming from a ×50 microscope objective (Mitutoyo) was reduced to ∼3–5 mW to protect the diamond anvils from failure because of the enhanced absorption of the highly stressed anvils. The typical exposure time was 60–120 s per Raman spectrum.

At each pressure point, the position of the X-ray beam was visualized with the X-ray-induced luminescence of the sample, and the spot was aligned relative to the center of a diamond culet, which was observed under illumination. For the Raman measurements, the focused laser beam was aligned relative to the image of the anvil. This alignment was reproducible at the same stage as the fixed DAC was placed in the Raman and X-ray setups with an accuracy of ∼1 µm. Thus, the X-ray and laser beams were aligned to probe the sample at the exact location. This is crucial because of the pressure gradients over the diamond tip. The original X-ray powder diffraction patterns of gold and the corresponding Raman spectra of stressed diamond anvils measured at different pressures in different runs are illustrated in Supplementary Fig. 1.

To measure the spatial pressure distribution at the diamond anvil tip, we performed X-ray diffraction and Raman mapping on an area of 20 × 20 µm^2^ with a horizontal and vertical step of 2 µm. At certain highest pressure points, the grid step was reduced to 0.5–1 µm. In addition, at selected pressure points, diamond anvils were scanned horizontally and vertically up to 70 μm away from the center of the diamond culet (see Supplementary Figs. 3–5, 7, and 8).

### Pressure determination

The diamond Raman edge was assigned to the pressure value estimated from the X-ray diffraction measurements by referring the refined lattice volume of the gold sample to the reduced isotherm obtained from the ramp experiments^23^. We used the mid-point of the cut-off of the Raman spectrum for the determination of pressure values in our samples. The mid-point of the step can be located with the accuracy of ∼2–6 cm^−1^ at pressures up to ∼400 GPa and ∼6–10 cm^−1^ at higher pressures that is equivalent to the uncertainty in a pressure value of ∼1–5.5 GPa and ∼5.5–10 GPa, respectively (see also Supplementary Table 1).

Quite often Raman spectra at high pressures have pronounce fringes (see Supplementary Fig. 1). To our knowledge they were not discussed before, however were observed by many groups. Apparently, this is not an interference of a luminescence as the fringes are observed only within the Raman band of the stressed diamond (1333 cm^−1^–∼2000 cm^−1^). Likely this is an interesting case when a source of light (the scattered Raman signal from the diamond) is within a cavity formed between the surface of the diamond culet and the edge of the stressed volume (see the inset in Fig. 1b). The latter is not a sharp boundary, however the light can be reflected from the wide layer characterized by a large gradient of the refractive index. This gradient may stem from the rapid change of the stresses inside the anvil as it was calculated in Refs. ^28,56^. The calculations show that the stressed volume propagates at ∼50 μm in depth for the typical diamond anvils at *P* ∼280 GPa^28,29^ (see the inset in Fig. 1b). This depth is consistent with the characteristic length derived from the interference patterns. In runs 1, 2, and 3 (where the maximum pressures were achieved) the interference fringes have a characteristic separation of ~50 cm^−1^. The interference can be described as 2*ndΔν* = 1, where *n* is a refractive index, *d* is a length where light interferes, and *Δν* is a separation between the neighbor fringes. Assuming that the refractive index of diamond deviates not too much from the ambient pressure value of *n* = 2.4, *d* can be roughly estimated as ~40 µm. This value approximately corresponds to the depth of the calculated stressed volume. The depth correlates with a diameter of the stressed volume, which depends on a diameter of the diamond culet. For 25 µm culets, the characteristic *Δν* = 15 cm^−1^ corresponds to a deeper stressed volume with a *d* ~130 µm. The interference pattern is complicated, the distance between the fringes is in fact unequal: it is maximum at the high-frequency edge and decreases towards the low-frequency edge of the diamond band. A detailed description of the interference requires the calculation of the spatial distribution of the refractive index. Probably it can be done on the basis of the detailed recent calculations of the stresses and strains in the loaded anvil^28^. Although the fringes complicate the observed Raman spectrum, they in fact do not prevent the determination of the diamond Raman edge because the fringes appear only within the diamond band (1333 cm^−1^–∼2000 cm^−1^) and the diamond Raman edge coincides with the first fringe.

The pressure values in Au samples were estimated from the X-ray diffraction data using the position of (111) diffraction peak, which is the least affected by non-hydrostatic compression at very high megabar pressures^2,35,36^. Such estimate is typically used for Au samples at megabar pressures and is in good agreement with more accurate equation of states of Au under comparable values of deviatoric stresses^2,35,36,57,58^. For example, the pressure values estimated in gold samples by this method in ref. ^2^ only slightly deviate from those determined from the ramp experiments^23^ (see Figs. 3e and 4 in ref. ^23^). The small difference in pressure values (up to ±5 GPa at ∼500 GPa) between two sets of data mainly stems from different equations of state of Au; the authors of ref. ^2^ used EoS of Au measured up to 123 GPa^35^ and extrapolated it to higher pressures.

The deviatoric stress *t* was estimated by applying the commonly used method^35,59,60^. This method is based on the analysis of the positions of the diffraction peaks observed in X-ray powder diffraction patterns because different peaks shift differently under non-hydrostatic stress. While in a cubic crystal lattice, all diffraction peaks yield identical lattice parameters, in the strained crystal they differ by a factor proportional to the difference between the maximal and minimal eigenvalues of the stress tensor and the elastic anisotropy of the crystal. For the cubic system, a lattice parameter refined from different diffraction peaks can be represented in the linear form by ${a}_{{hkl}}={M}_{o}+{M}_{1}[3{{\Gamma }}_{{hkl}}(1-3{{{\sin }}}^{2}{\theta }_{h{kl}})]$, where *M*_*0*_ and *M*_*1*_ are fitting parameters, ${{\Gamma }}_{{hkl}}=\frac{({h}^{2}{k}^{2}+{k}^{2}{l}^{2}+{l}^{2}{h}^{2})}{{({h}^{2}+{k}^{2}+{l}^{2})}^{2}}$, *θ*_*hkl*_ is a diffraction angle and *h*, *k*, *l* are Miller indices of diffraction peaks. And the deviatoric stress can be estimated by $t\cong -\frac{3{M}_{1}}{\alpha S{M}_{0}}$. Parameter *α*, which varies between 0 (iso-strain assumption) and 1 (iso-stress assumption), was set to 1 based on observations from radial X-ray diffraction measurements, which proved that α remains close to 1 under high pressure^61^. The elastic anisotropy parameter of the crystal *S* was calculated by equation $S=\frac{C-C{\prime} }{2{CC}{\prime} }$, where *C* = *C*_*44*_ and *C’* = (*C*_*11*_-*C*_*12*_)/2 are the two share constants of the cubic crystal. Isothermal elastic constants of gold and their pressure derivatives were taken from ref. ^62^ We analyzed the deviation of lattice parameters refined from different observed diffraction peaks in measured X-ray diffraction patterns of Au and plotted it as Γ-plots in Supplementary Fig. 9. In most our runs the estimated deviatoric stress reaches ∼10–15 GPa, i.e. ∼2–3% at highest pressures (see Fig. 3b), which is comparable with that estimated for Au samples compressed up to ∼600 GPa in toroidal diamond anvils^2^ but less than that sustained in the ramp experiments^23^.

## Supplementary information

Supplementary information

## Data Availability

The data that support the findings of this study are also available from the corresponding author upon request. Source data are provided with this paper.

## Source data

Source Data
