# Supplementary material for: Universal diamond edge Raman scale to 0.5 terapascal and implications for the metallization of hydrogen
Source: Nat Commun. 2023 Feb 17;14:907. doi: 10.1038/s41467-023-36429-9 (PMC9938121; doi:10.1038/s41467-023-36429-9)
Supplement: Supplementary file 1 — Supplementary information [file 41467_2023_36429_MOESM1_ESM.pdf]

Supplementary Information

Universal diamond edge Raman scale to 0.5 terapascal and implications for the metallization of hydrogen

M. I. Eremets[1]*, V. S. Minkov[1], P. P. Kong[1], A. P. Drozdov[1], S. Chariton[2], V. B. Prakapenka[2]

[1]*Max Planck Institute for Chemistry; Hahn Meitner Weg 1, Mainz, 55128, Germany*
[2]*Center for Advanced Radiation Sources, University of Chicago; 5640 South Ellis Avenue, Chicago, Illinois, 60637, USA*

*Corresponding author. Email: m.eremets@mpic.de

Supplementary Figures 1 to 9
Supplementary Table 1
Supplementary references

SUPPLEMENTARY FIGURES

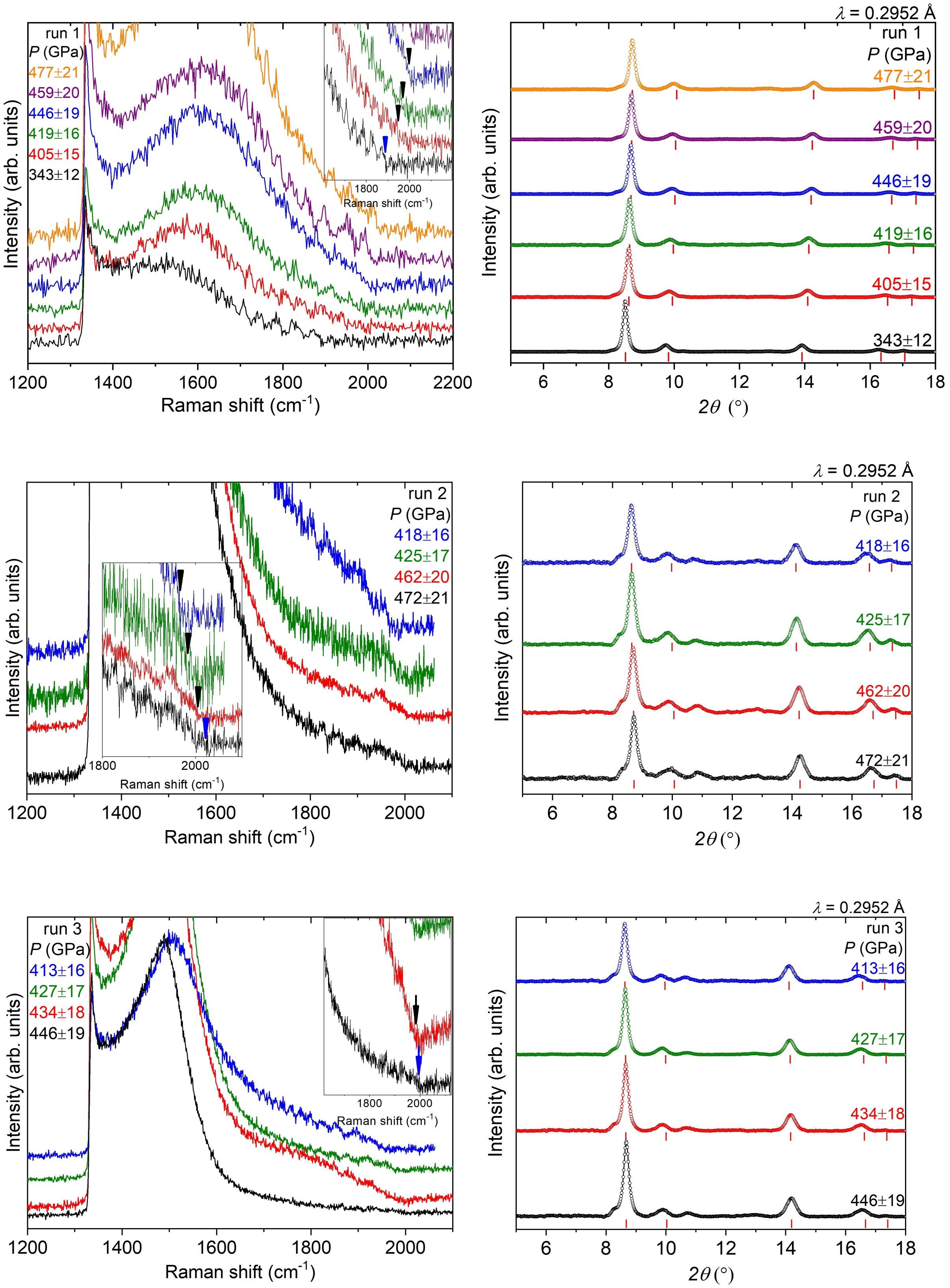

Supplementary Figure 1. Original Raman spectra of stressed diamond anvils (left) and corresponding X-ray diffraction powder patterns of gold samples (right) measured in runs 1-8 at different loads. Red ticks correspond to the calculated positions of diffraction peaks of Au at estimated pressure values according to the equation of state of Au[1]. Insets in the Raman plots show the diamond Raman edge in more detail, arrows indicate the middle of the steps. Source data are provided as a Source Data file.

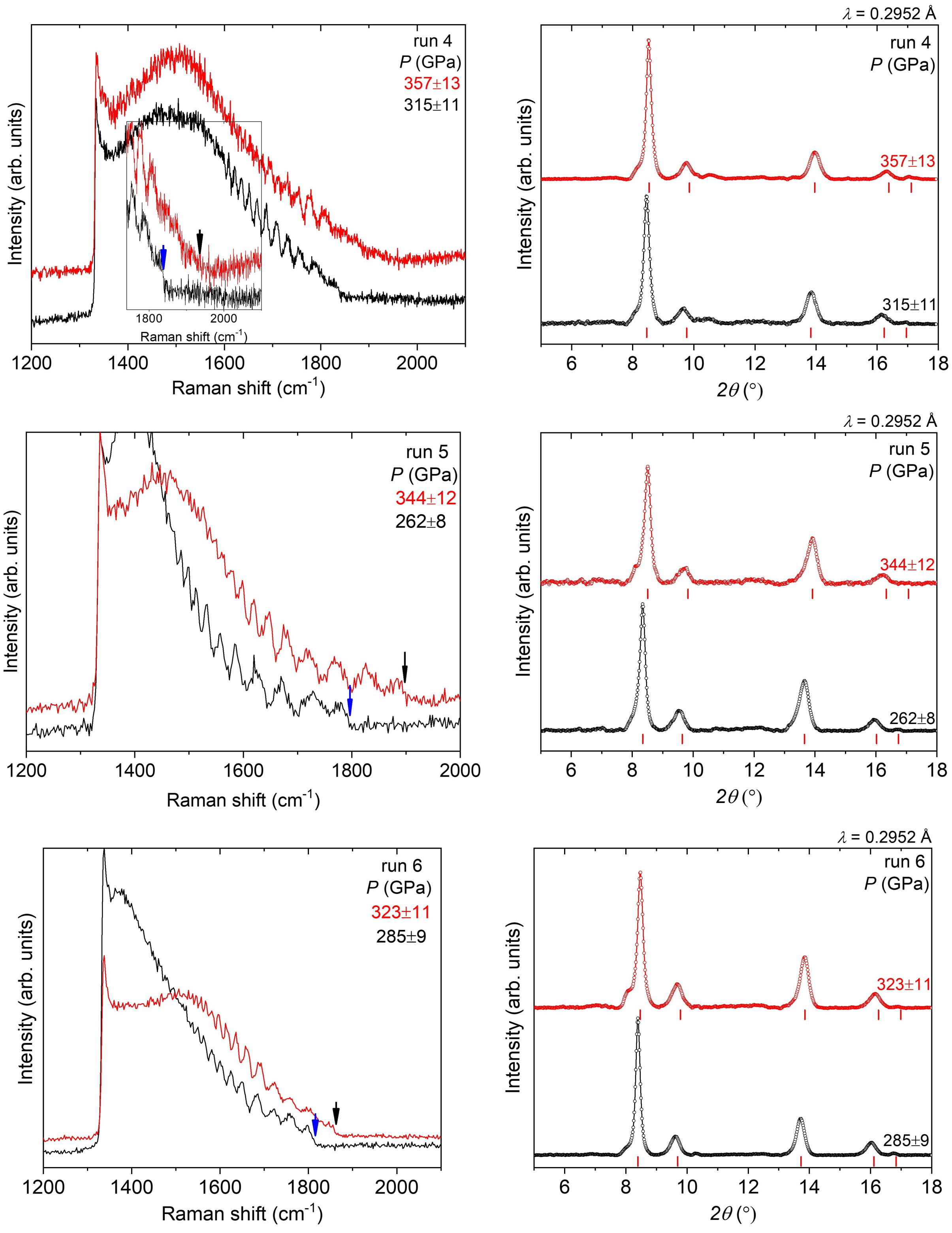

Supplementary Figure 1 (continuation). Original Raman spectra of stressed diamond anvils (left) and corresponding X-ray diffraction powder patterns of gold samples (right) measured in runs 1-8 at different loads. Red ticks correspond to the calculated positions of diffraction peaks of Au at estimated pressure values according to the equation of state of Au[1]. Insets in the Raman plots show the diamond Raman edge in more detail, arrows indicate the middle of the steps. Source data are provided as a Source Data file.

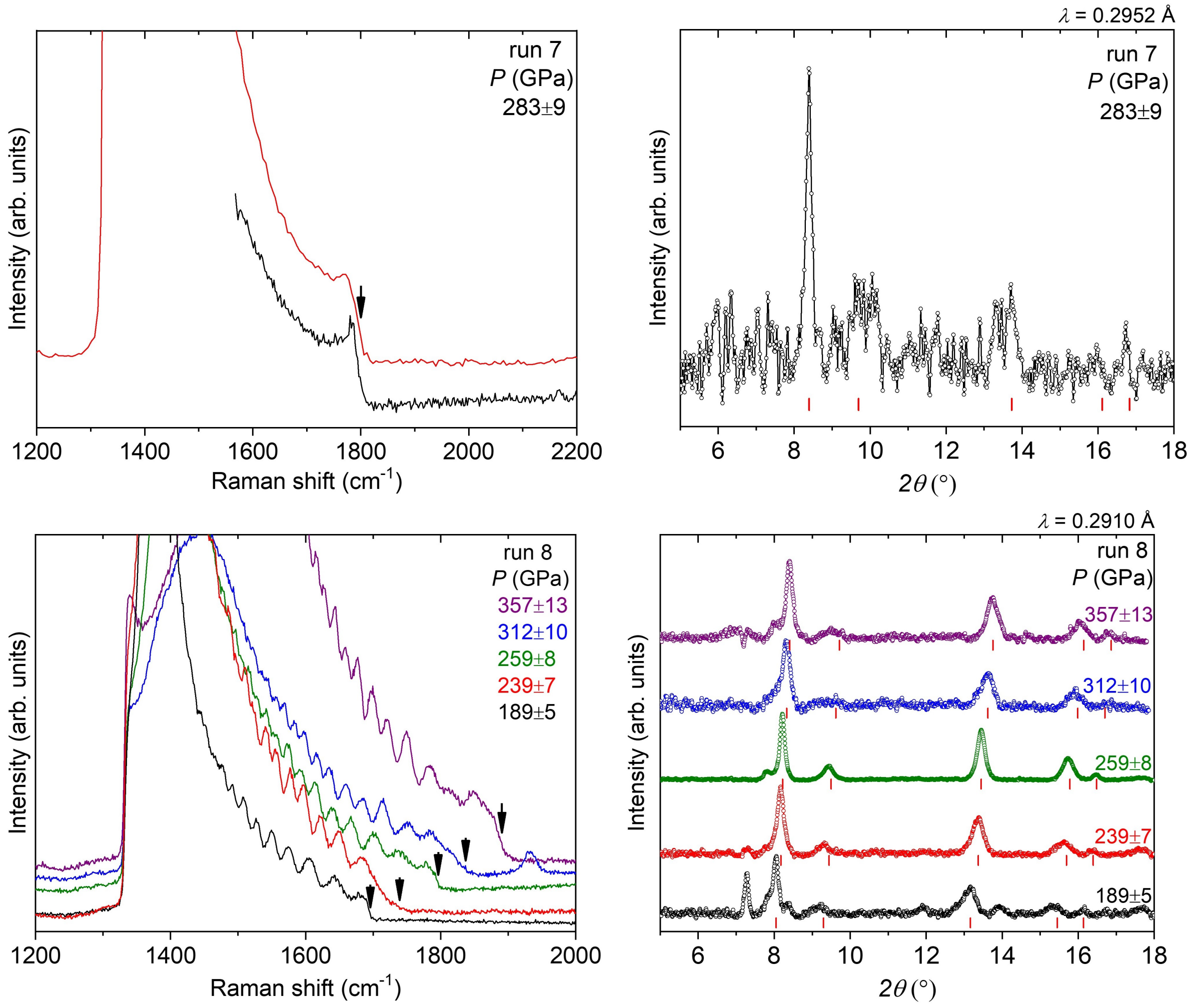

Supplementary Figure 1 (continuation). Original Raman spectra of stressed diamond anvils (left) and corresponding X-ray diffraction powder patterns of gold samples (right) measured in runs 1-8 at different loads. Red ticks correspond to the calculated positions of diffraction peaks of Au at estimated pressure values according to the equation of state of Au[1]. Insets in the Raman plots show the diamond Raman edge in more detail, arrows indicate the middle of the steps. Source data are provided as a Source Data file.

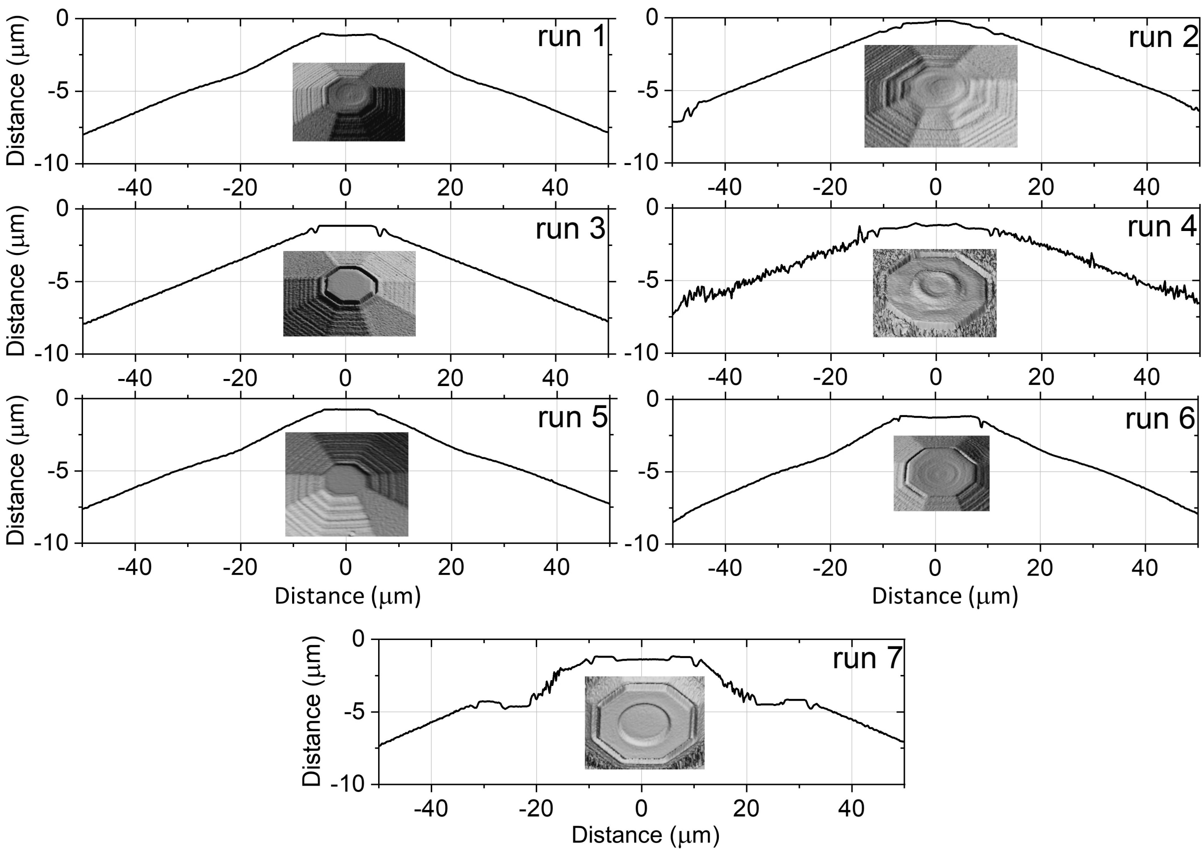

Supplementary Figure 2. Profiles of diamond anvil tips used in runs 1-7. Insets show 3D image of the culets in the same scale. In run 8 we did not measure a profile, in this run anvils had 8-μm-diameter culets beveled at 7.5° to a diameter of 320 μm. Source data are provided as a Source Data file.

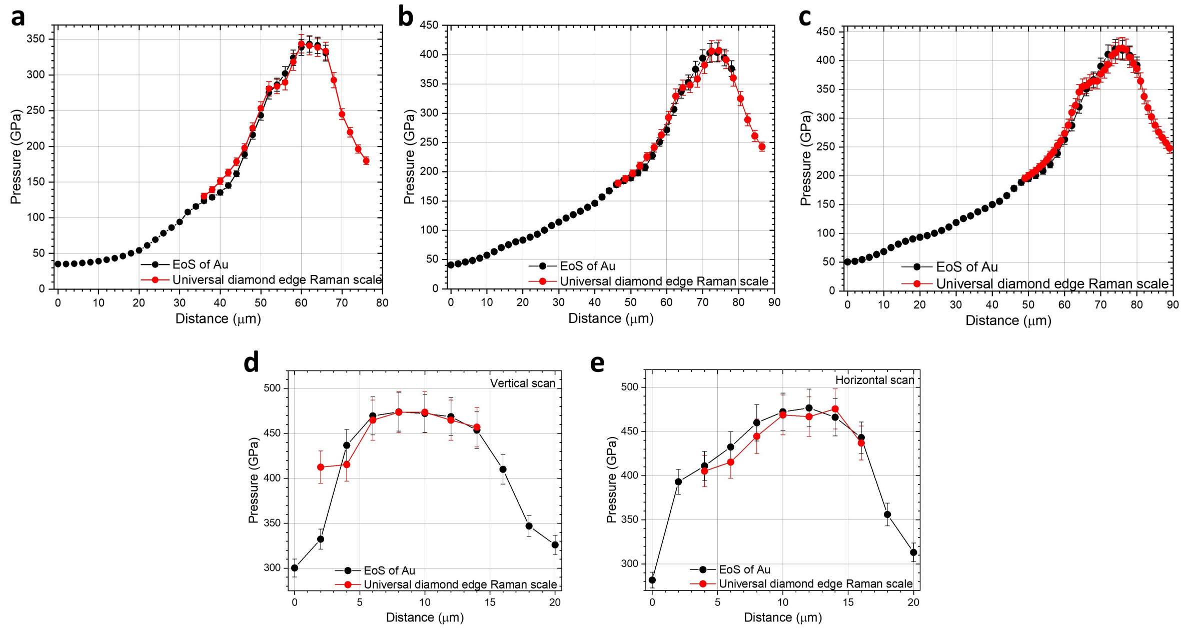

Supplementary Figure 3. Distribution of pressure on the diamond tip in run 1 at several loads. (**a** – **c**) Pressure distribution plotted from one-dimensional scans: X-ray diffraction (black circles) and Raman (red circles). (**d** and **e**) Pressure distribution on the diamond culet at the highest load. Note that the present universal diamond edge Raman scale which was built on the measurements at the points at maximum of pressures works also at the large area – pressure values well consist with the pressure determined with the gold gauge (equation of state of Au[1] (X-ray diffraction)). Source data are provided as a Source Data file.

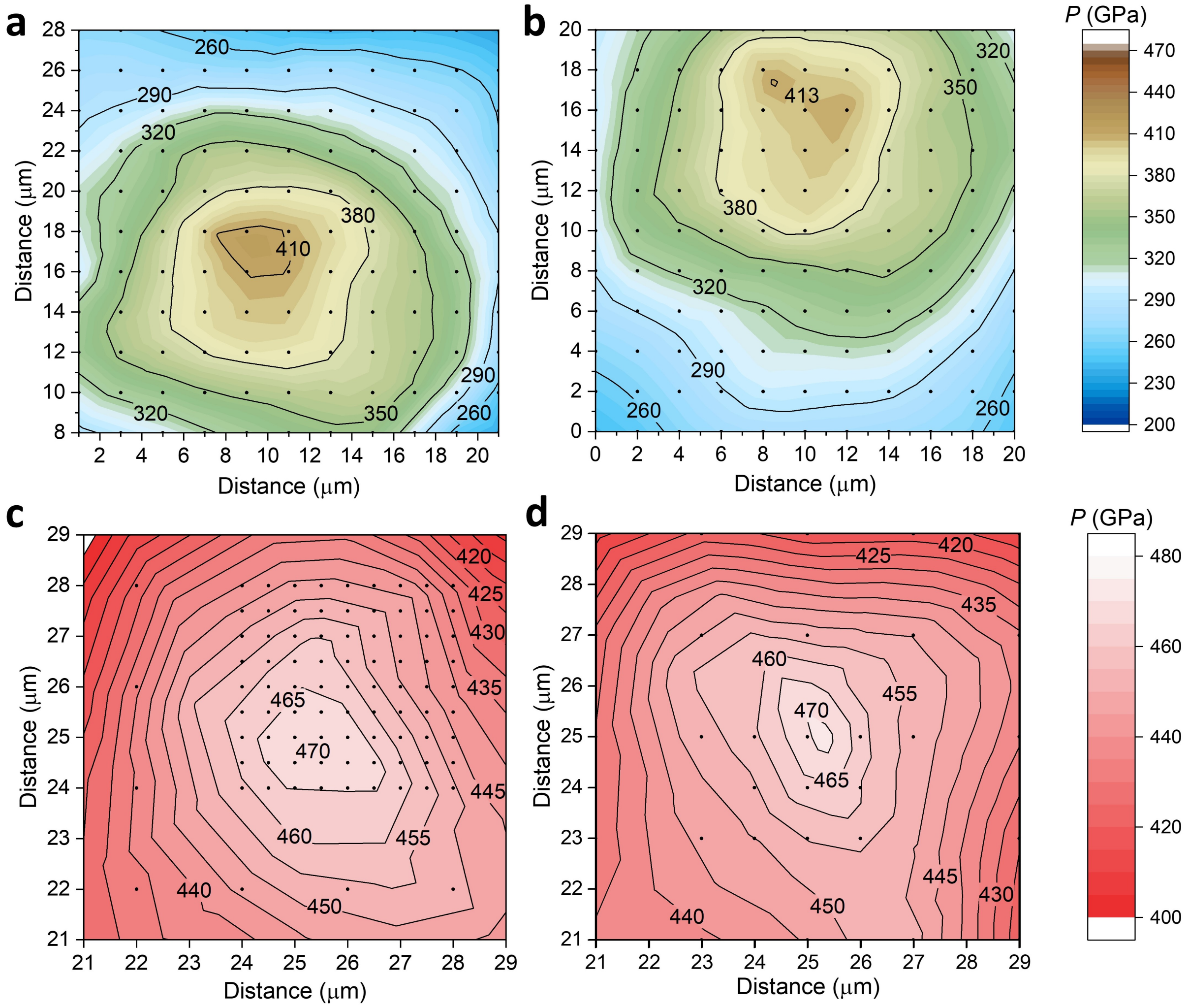

Supplementary Figure 4. Spatial distribution of pressure on the diamond tip in run 2 at several loads. (**a** and **b**) Extended pressure distribution plots reconstructed from two-dimensional X-ray diffraction and Raman mappings. (**c** and **d**) Plots of pressure distribution on the diamond culet at the highest load. Pressure values were estimated using the equation of state of Au[1] (X-ray diffraction, panels **a** and **c**) and the universal diamond edge Raman scale (panels **b** and **d**). Black points are spots of measurements. Source data are provided as a Source Data file.

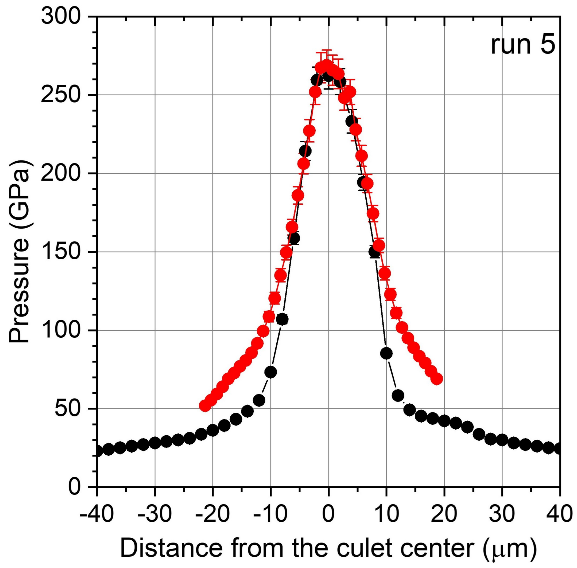

Supplementary Figure 5. Distribution of pressure on the diamond tip (culet 10 μm) in run 5. The plot is reconstructed from one-dimensional scans: X-ray diffraction (black circles) and Raman (red circles). Pressure values were estimated using the equation of state of Au[1] (X-ray diffraction) and the universal diamond edge Raman scale. The difference in pressures values at distances farther ~10 μm from the diamond culet center is naturally expected as the pattern of stresses in the anvil strongly changes[2]. However, the pressure values well coincide within the diamond culet area. This fact supports the universality of the present pressure scale. Source data are provided as a Source Data file.

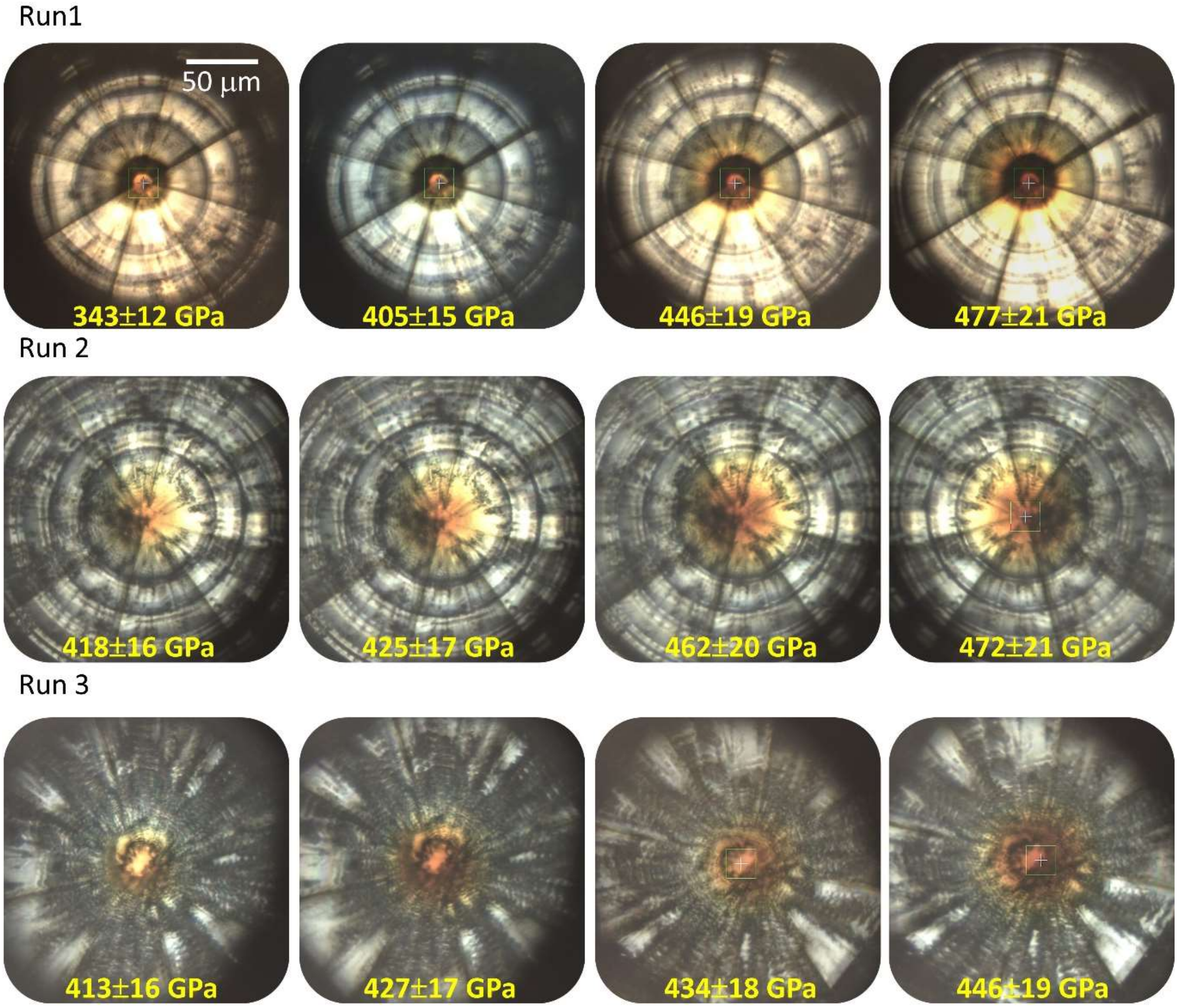

Supplementary Figure 6. Photos of diamond anvils with gold samples at highest achieved pressures in runs 1-3. Diamond anvils become darker with pressure: the bronze-colored area near the diamond culet saturates and propagates towards diamond bevels. Pressure values are estimated using the equation of state of Au[1].

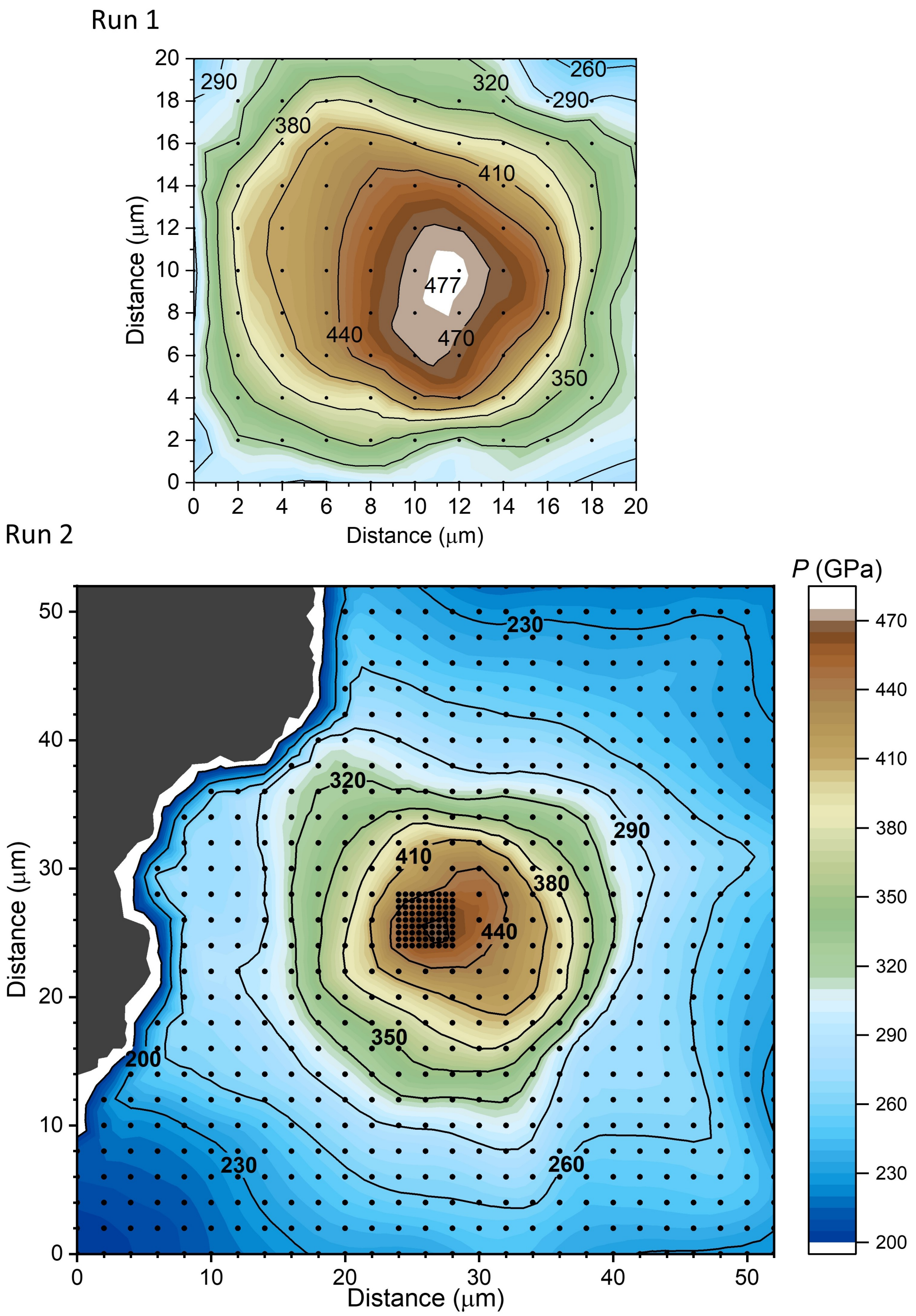

Supplementary Figure 7. Spatial distribution of pressure on the diamond tip in run 1 and 2 at maximum loads. The plots are reconstructed from two-dimensional X-ray diffraction mappings. Black points are spots of measurements. Pressure values are estimated using the equation of state of Au[1]. Source data are provided as a Source Data file.

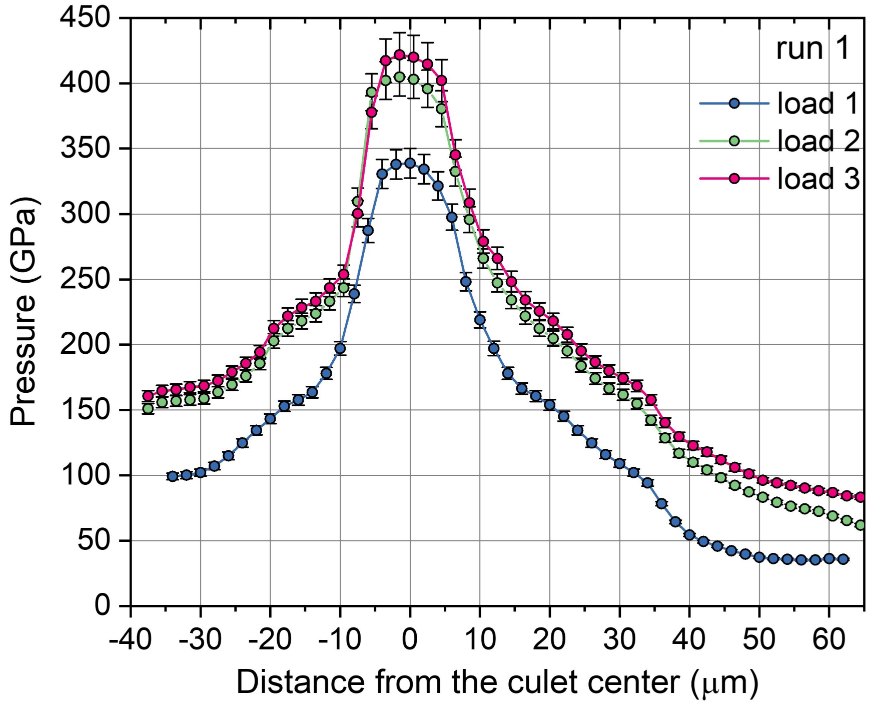

Supplementary Figure 8. Distribution of pressure on the diamond tip in run 1 at several loads. The plot demonstrates in particular that the pressure is concentrated in the area close to the culet, its size is ~10 μm. Plots are reconstructed from one-dimensional X-ray diffraction scans. Pressure values are estimated using the equation of state of Au[1]. Source data are provided as a Source Data file.

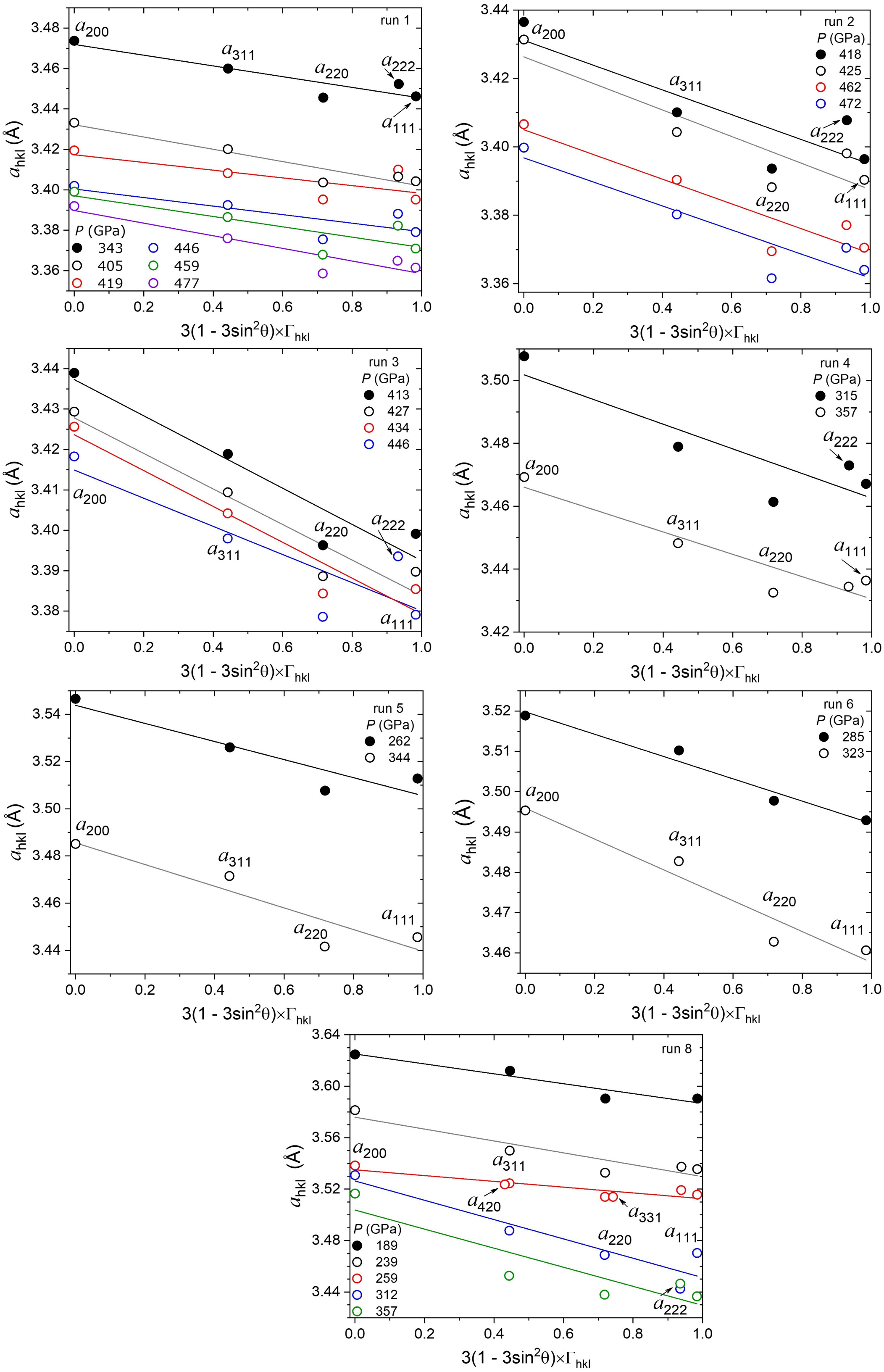

Supplementary Figure 9. Representative Γ-plots of the observed diffraction peaks of Au in run 1-6 and 8 at different loads. Solid lines represent the linear fit of lattice parameters derived from different diffraction peaks using equation $a_{hkl} = M_o + M_1[3\Gamma_{hkl}(1 - 3\sin^2\theta_{hkl})]$ (see details in Methods). Source data are provided as a Source Data file.

Supplementary Table 1. Propagation of uncertainties for the universal diamond edge Raman scale. Uncertainty contributions for determining the sample pressure in diamond anvil cells using the Raman edge of the stressed diamond anvil. Uncertainties in estimated pressure values associated with different sources are included (ΔP_1 – from the EoS of Au[1]; ΔP_2 – from uncertainty in determination of diamond Raman edge). The resulting ΔP_{total} is estimated as $\sqrt{(\Delta P_1)^2 + (\Delta P_2)^2}$. Source data are provided as a Source Data file.

Wavenumber of diamond Raman edge (cm^{-1})	Pressure (GPa)	$\pm\Delta P_1$ (GPa)	$\pm\Delta P_2$ (GPa)	$\pm\Delta P_{total}$ (GPa)
1334	0.4	0.0	0.8	0.8
1337	1.8	0.1	0.8	0.8
1341	3.4	0.2	0.8	0.8
1351	7.3	0.3	0.8	0.9
1356	9.3	0.3	0.8	0.9
1361	11.5	0.4	0.8	0.9
1367	13.8	0.4	0.8	0.9
1373	16.5	0.4	0.8	0.9
1379	18.8	0.5	0.9	1.0
1385	21.5	0.5	0.9	1.0
1391	24.2	0.5	0.9	1.0
1397	27.0	0.6	0.9	1.1
1404	29.8	0.6	0.9	1.1
1411	33.1	0.7	0.9	1.1
1417	36.1	0.7	0.9	1.2
1425	39.6	0.8	0.9	1.2
1432	42.9	0.8	0.9	1.2
1440	46.5	0.9	1.0	1.3
1447	50.2	1.0	1.0	1.4
1455	53.9	1.1	1.0	1.5
1463	58.0	1.2	1.0	1.6
1471	61.9	1.3	1.0	1.6
1479	66.1	1.4	1.0	1.7
1487	70.4	1.6	1.0	1.9
1496	74.8	1.7	1.1	2.0
1504	79.2	1.9	1.1	2.2
1513	84.1	2.0	1.1	2.3
1522	89.0	2.1	1.1	2.4
1531	94.0	2.3	1.1	2.6
1540	99.1	2.4	1.1	2.7
1549	104.2	2.5	1.1	2.8
1558	109.4	2.7	2.3	3.6
1567	114.7	2.9	2.4	3.7
1577	120.4	3.1	2.4	3.9
1587	126.3	3.2	2.4	4.0
1596	132.2	3.3	2.5	4.1
1606	138.2	3.5	2.5	4.3
1616	144.3	3.6	2.5	4.4

Wavenumber of diamond Raman edge (cm^{-1})	Pressure (GPa)	$\pm\Delta P_1$ (GPa)	$\pm\Delta P_2$ (GPa)	$\pm\Delta P_{total}$ (GPa)
1625	150.4	3.8	2.6	4.6
1635	156.7	4.0	2.6	4.8
1646	163.5	4.1	2.6	4.9
1655	169.9	4.3	2.7	5.1
1666	176.9	4.5	2.7	5.2
1675	183.4	4.8	2.7	5.5
1686	190.8	5.0	2.8	5.7
1696	197.8	5.3	2.8	6.0
1707	205.1	5.6	2.8	6.3
1717	212.6	5.9	2.9	6.6
1728	220.6	6.2	2.9	6.9
1749	236.4	6.9	3.0	7.5
1760	244.3	7.2	3.0	7.8
1771	252.6	7.6	3.1	8.2
1782	261.2	8.0	3.1	8.6
1793	269.8	8.4	4.7	9.6
1804	278.6	8.8	4.8	10.0
1815	287.4	9.2	4.8	10.4
1826	296.4	9.6	4.9	10.8
1837	305.4	10.0	4.9	11.2
1849	314.6	10.5	5.0	11.6
1860	324.4	10.9	5.1	12.0
1871	333.8	11.4	5.1	12.5
1883	343.3	11.9	5.2	13.0
1894	353.5	12.4	5.2	13.5
1905	363.2	12.9	5.3	13.9
1916	373.1	13.5	5.4	14.5
1928	383.6	14.2	5.4	15.2
1939	393.6	14.8	5.5	15.8
1965	417.2	16.4	5.6	17.3
1973	424.7	17.0	7.5	18.6
1985	436.0	18.0	7.6	19.5
1996	446.5	19.0	7.7	20.5
2011	461.0	20.2	7.8	21.7
2026	476.7	21.4	10.0	23.6

SUPPLEMENTARY REFERENCES

1. Fratanduono, D. E. *et al.* Establishing gold and platinum standards to 1 terapascal using shockless compression. *Science* **372**, 1063–1068 (2021).
2. Adams, D. M. & Shaw, A. C. A computer-aided design study of the behaviour of diamond anvils under stress. *J. Phys. D: Appl. Phys.* **15**, 1609 (1982).
